# Direct selective growth of ZnO nanowire arrays from inkjet-printed zinc acetate precursor on a heated substrate

**DOI:** 10.1186/1556-276X-8-489

**Published:** 2013-11-20

**Authors:** Jinhyeong Kwon, Sukjoon Hong, Habeom Lee, Junyeob Yeo, Seung S Lee, Seung Hwan Ko

**Affiliations:** 1 Department of Mechanical Engineering, KAIST, 291 Daehak-ro, Yusong-Gu, Daejeon 305-701, South Korea; 2 Department of Mechanical Engineering, Seoul National University, Seoul, 1 Gwanak-ro, Gwanak-gu, Seoul 151-742, South Korea

**Keywords:** Inkjet printing, ZnO nanowire, Direct patterned growth, Hydrothermal growth, Network transistors, UV sensors

## Abstract

Inkjet printing of functional materials has drawn tremendous interest as an alternative to the conventional photolithography-based microelectronics fabrication process development. We introduce direct selective nanowire array growth by inkjet printing of Zn acetate precursor ink patterning and subsequent hydrothermal ZnO local growth without nozzle clogging problem which frequently happens in nanoparticle inkjet printing. The proposed process can directly grow ZnO nanowires in any arbitrary patterned shape, and it is basically very fast, low cost, environmentally benign, and low temperature. Therefore, Zn acetate precursor inkjet printing-based direct nanowire local growth is expected to give extremely high flexibility in nanomaterial patterning for high-performance electronics fabrication especially at the development stage. As a proof of concept of the proposed method, ZnO nanowire network-based field effect transistors and ultraviolet photo-detectors were demonstrated by direct patterned grown ZnO nanowires as active layer.

## Background

Recently, there has been a tremendous interest in 3D printing which is one of research branches in additive direct printing approach of functional materials. Additive direct printing method has relatively shorter history compared with conventional photolithography- and vacuum deposition-based microelectronics fabrication processes. Direct printing method has made dramatic progress with the invention of drop-on-demand (DOD) inkjet printer and has gained significant interest as an alternative to conventional integrated circuit (IC) process especially in the area of low-cost flexible electronics [1-3]. Conventional photolithography-based processes are basically subtractive approach which wastes most of the expensive materials away during the process, and so, they are hard to accommodate any changes during the process. Furthermore, conventional IC processes involve multistep; therefore, they are very time consuming and expensive. In this regard, the DOD inkjet printing as an additive process has drawn tremendous attention because inkjet printing is fully data driven and maskless process which allows more versatility than other direct printing methods. The material is deposited in a carrier solution on the substrate by a piezo-electrically driven micro capillary tube. This solution processing provides high flexibility for choosing both the depositing material and the substrate [1].

The inkjet printing method opened a new research area in the direct nanomaterial manipulation on the predetermined locations with a controlled morphology and a specific location of nanoparticles [4-6] and nanowires [7,8], and more recently, direct local nanowire growth by seed nanoparticle inkjet printing has been demonstrated by Ko et al. [9]. Conventional nanomaterial manipulation uses a series of multisteps for growth, harvest, and placement of nanowires, which are very time consuming, expensive, and low yield. Inkjet printing of nanomaterials could overcome the difficulties encountered in multi-step serial processes, new approaches use the direct growth at specific location with desired nanowire morphology. However, direct inkjet printing of nanoparticle or nanowires has a fundamental drawback in inkjet nozzle clogging and limited ink choice in concentration and viscosity.

In this research, we introduce direct selective nanowire array growth by inkjet printing of Zn acetate precursor ink patterning and subsequent hydrothermal ZnO local growth without using ZnO nanoparticle seed to remove frequent nozzle clogging problem and without using conventional multistep processes. The proposed process can directly grow ZnO nanowire in any arbitrary patterned shape and it is basically very fast, low cost, environmentally benign, and low temperature. Therefore, zinc acetate precursor inkjet printing-based direct nanowire local growth is expected to give extremely high flexibility in nanomaterial patterning for high-performance electronics fabrication especially at the development stage. As a proof of concept of the proposed method, ZnO nanowire network-based field effect transistors and ultraviolet (UV) photodetectors were demonstrated by direct patterned grown ZnO nanowires as active layer.

## Methods

**Figure 1:**
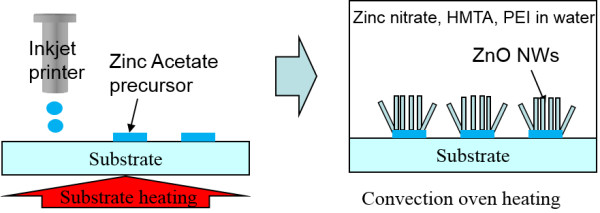
**Process schematics of the direct patterned ZnO nanowire growth from the inkjet-printed Zn acetate patterns.** After Zn acetate inkjet printing, ZnO nanowires were grown hydrothermally at 90°C heating for 2.5 h.

ZnO nanowire arrays were selectively grown from the inkjet-printed Zn acetate on glass or Si wafer through the hydrothermal decomposition of a zinc complex. The process is mainly composed of two simple steps as shown in Figure 1; (1) Zn acetate inkjet printing and thermal decomposition on a substrate, and (2) subsequent selective ZnO nanowire hydrothermal growth on the inkjet-printed Zn acetate patterns.

### Zn acetate ink for seed layer generation

For general ZnO nanowire growth, spin coating [10,11] or inkjet printing [9] of ZnO nanoparticle solution has been usually used as seed layer preparation. Instead of using nanoparticle seeds, in this research, Zn acetate precursor ink was inkjet printed for the local growth of ZnO nanowire arrays. While ZnO nanoparticle solution causes inkjet nozzle clogging problem, Zn acetate precursor ink can remove that problem completely. The Zn acetate ink was prepared from 5 mM zinc acetate (C_4_H_6_O_4_Zn, Sigma Aldrich, St. Louis, MO, USA) in ethanol. The Zn acetate ink was inkjet printed on the heated target substrate. The dried Zn acetate is thermally decomposed (200°C to 350°C for 20 min) to fine ZnO quantum dots as ZnO nanowire seeds. Thermal decomposition step in the air converts Zn acetate into uniform ZnO nanoparticles as well as promotes the adhesion of ZnO seed nanoparticles to the substrate. Alternatively, this thermal decomposition step may be done selectively by focused laser scanning [12].

### Zn acetate inkjet printing

Instead of spin coating on the whole substrate, inkjet printing method was used to locally deposit and pattern the seed layer. The Zn acetate solution was inkjet printed by a piezo-electrically driven DOD inkjet head integrated with CAD system to draw arbitrary patterns of Zn acetate ink. The 50-μm-sized droplets could be generated by changing nozzle diameter, jetting parameter (applied voltage waveform and amplitude) from the nozzle. The final printed droplet pattern size is adjusted by the substrate heating condition. The detailed jetting system set up and jetting parameters can be found in [9,12].

### ZnO NW selective growth

As shown in Figure 1, ZnO NWs were selectively grown only on the inkjet-printed Zn acetate patterns. The Zn acetate-printed and thermally decomposed patterns on the substrate are immersed in aqueous solutions containing 25 mM zinc nitrate hydrate, 25 mM hexamethylenetetramine (HMTA), and 5 to 7 mM polyethylenimine (PEI, branched, low molecular weight) at 90°C for 2.5 h to selectively grown ZnO arrays. Conventional solution-grown ZnO nanowire arrays have been limited to aspect ratios of less than 20. However, addition of PEI could boost the aspect ratio of ZnO NW above 125 by hindering only the lateral growth of the nanowires in solution while maintaining a relatively high nanowire density [11]. The substrate was placed upside-down to remove the unexpected precipitation of homogeneously grown ZnO NW on the substrate in an open crystallizing dish filled with solutions. Additionally, a thin cover glass was placed on the substrate with 2-mm spacer to control and suppress the natural convection and the subsequent byproduct growth on the unpatterned (unseeded) adjacent substrate region. Finally, the ZnO NWs grown on the substrate were thoroughly rinsed with MilliQ water (Millipore Corporation, Billerica, MA, USA) and dried in air at 120°C to remove any residual solvent and optimize the electrical performance.

### ZnO nanowire network transistor and UV sensor fabrication and characterization

Selective ZnO growth from the inkjet-printed Zn acetate pattern can be applied to various ZnO nanowire-based functional device demonstration. In this research, ZnO nanowire network transistors (NWNT) [13] as active layer for the transistor and ZnO UV sensor by local growth on ZnO nanowire network were demonstrated. The ZnO NWNT fabricated in this work have a bottom gate/bottom contact configuration wherein the channel length is defined by the separation between the two parallel electrodes (source and drain) on top of SiO_2_/n + Si wafer back gate. Photolithographically patterned gold source and drain electrodes are connected by the network path composed of numerous 1- to 3-μm ZnO NW [13]. The ZnO UV sensor also has similar structures but without back gate. ZnO nanowires were locally grown on the Zn acetate inkjet-printed area in the gap between two adjacent metal electrode pads. The photoconductive UV sensor changes the conductivity of ZnO crystal upon the UV light irradiation.

The transistor performance (transfer and out characteristics) was characterized using a HP4155A semiconductor parameter analyzer (Agilent technologies, Santa Clara, CA, USA) in a dark Faraday cage in air. The photoconductivity of ZnO nanowire UV sensor was investigated through the transient current change measurement under UV light with a fixed bias of 1 V. For UV illumination, a UV lamp with the center wavelength at 365 nm is turned on and off alternatively for every 100 s.

## Results and discussion

**Figure 2:**
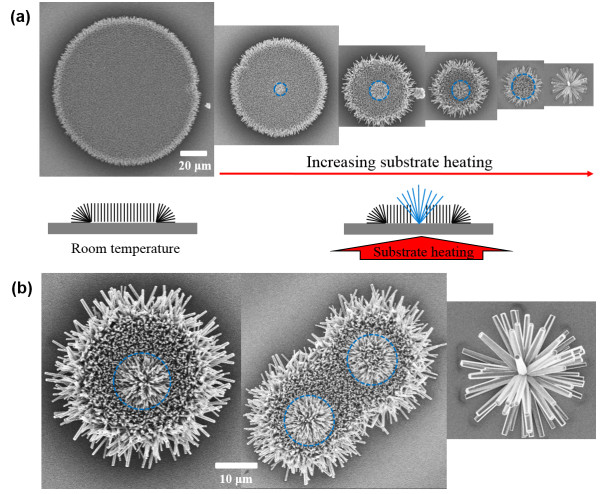
**SEM pictures of the hydrothermally grown ZnO nanowire array on the inkjet-printed Zn acetate patterns. (a)** ZnO nanowire array size variation at increased substrate heating; room temperature, 30°C, 40°C, 50°C, 60°C, and 70°C heating from left to right. Inset schematics show the cross sectional view of the ZnO nanowire array. **(b)** Magnified SEM pictures of 50°C, 60°C, and 70°C from left to right. Blue dotted lines indicate the elevated ZnO array at the center of the droplet due to substrate heating.

Figure 2 show the SEM (scanning electron microscope) images of selectively grown ZnO nanowire array on the inkjet-printed Zn acetate droplets. The ZnO nanowires grew only on the Zn acetate printed patterned. The initial printed droplet size of the Zn acetate precursor was 100 to 120 μm in diameter at room temperature. The usual length of the individual ZnO nanowire was around 1 to 3 μm with 100 to 150 nm in diameter after one time growth and longer nanowire could be obtained by introducing the samples repeatedly into fresh solution baths every several hours. ZnO nanowires have hexagonal cross sections and grow along the *c*-axis of the wurtzite crystal in the [0001] direction. Bottom inset schematics show the cross-sectional view of the grown ZnO nanowire array. The ZnO nanowire arrays are grown vertically within ±10° deviation angle on the central part of a circular pattern while urchin-like nanowires are grown at the edge of the circular pattern. The urchin-like dense ZnO NWs show highly ordered outward radial directional growth because urchin-like radial growth minimizes the interaction among each nanowires and the affluent precursor supply from outside of the circular seed pattern redirects the nanowire growth to the outward direction compared with the central part [9].

The inkjet print head with 50-μm-diameter nozzle originally generated 50-μm Zn acetate ink droplets, and they spread out and dried to various sized circular pattern depending on the substrate heating condition. Substrate heating can reduce the spreading of the Zn acetate ink. Figure 2a shows that the grown ZnO array size can be adjusted by substrate heating from room temperature to 70°C (room temperature, 30°C, 40°C, 50°C, 60°C, 70°C, respectively from left). The inkjet-printed precursor droplet will dry on the substrate. Substrate heating will accelerate the drying rate and subsequently increase contact line receding rate as the heating temperature increases. At high drying rate, the contact line will recede to smaller pattern to reduce to the size of the grown ZnO nanowire array. As the heating temperature increases, elevated ZnO nanowires were observed at the center of the droplet as indicated as blue dotted lines in Figure 1. The elevated ZnO nanowires were not observed in ZnO nanowire array grown from the nanoparticle inkjet printing [9]. The elevated ZnO nanowires might be due to the high concentration of the Zn acetate precursor during the fast drying process on the heated substrate. At the extreme cases, Zn acetate ink droplet may shrink to the size of the single nanowire diameter size to grow a single ZnO nanowire. However, the smallest nanowire array was a bundle of nanowire array growing from a point as shown in Figure 2b (left figure) at 70°C substrate heating case. For that case, the nanowire diameter and length were much bigger than those of the nanowires grown from the larger inkjet patterns. Interestingly, when two droplets have overlap, the grown ZnO nanowire array has little influence to each other.

Nanowires have been used for next generation high-performance electronics fabrication. For functional nanowire-based electronics fabrication, conventionally, combination of complex multiple steps, such as chemical vapor deposition growth of nanowire, harvesting of nanowire, manipulation and placement of individual nanowires, and integration of nanowire to circuit are necessary [14]. Each step is very time consuming, expensive, and environmentally unfriendly, and only a very low yield is achieved through the multiple steps. However, direct local growth of the nanowires from the inkjet-printed Zn acetate precursor can be used as a good alternative to the conventional complex multistep approach by removing multiple steps for growth, harvest, manipulation/placement, and integration of the nanowires. The ease and simplicity of current process even can allow using the household desktop inkjet printer.

**Figure 3:**
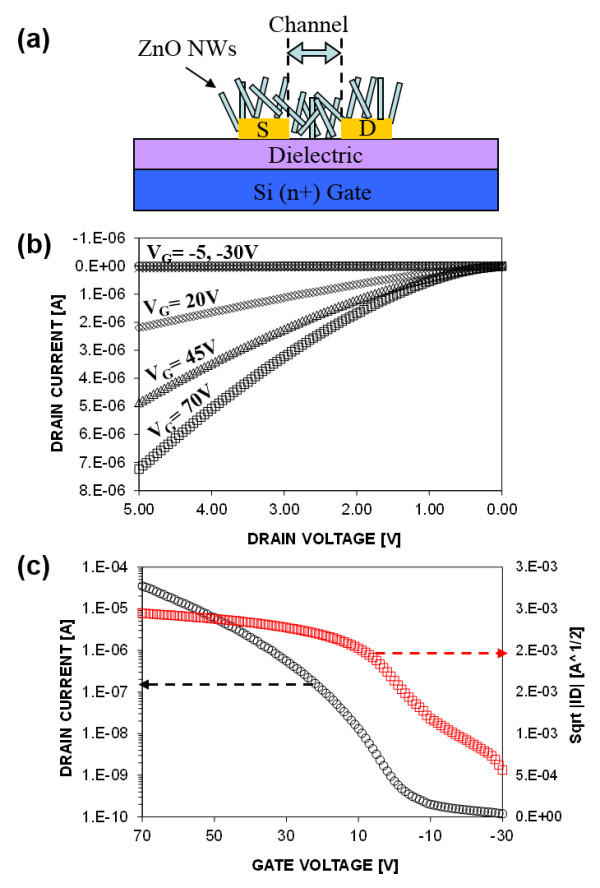
**ZnO nanowire network transistor demonstration. (a)** Schematic illustration of the transistor. ‘S’ and ‘D’ indicate source and drain electrodes, respectively. **(b)** Output and **(c)** transfer characteristics of the ZnO NWNT with 10-μm channel length. For output characteristics measurement, the drain voltage (*V*_d_) was scanned from 0 to 5 V and the drain current (*I*_d_) was measured while the gate voltage (*V*_g_) was fixed at -30, -5, 20, 45, and 70 V during each *V*_d_ scanning. *V*_g_ was scanned from -30 to 70 V and the drain current (*I*_d_) was measured while *V*_d_ was fixed at 5 V for transfer characteristics measurement.

Current proposed approach was applied to demonstrate ZnO NWNT by local growth on ZnO nanowire network as active layer for the transistor. The ZnO nanowires were selectively grown on the inkjet-printed Zn acetate pattern. The network path is composed of numerous 1- to 3-μm ZnO NWs connecting the source and drain electrodes (Figure 3a). The output and transfer characteristics of the ZnO NWNT are shown in Figure 3b,c for 10-μm channel length. For output characteristics measurement (Figure 3b), the drain voltage (*V*_d_) was scanned from 0 to 5 V and the drain current (*I*_d_) was measured while the gate voltage (*V*_g_) was fixed at -30, -5, 20, 45, and 70 V during each *V*_d_ scanning. *V*_g_ was scanned from -30 to 70 V and the drain current (*I*_d_) was measured while *V*_d_ was fixed at 5 V for transfer characteristics measurement (Figure 3c). The fabricated ZnO NWNT shows typical operation in n-type accumulation device characteristics working in a depletion mode [13]. The effective field effect mobility (***μ***_FE_) with 100% coverage assumption was calculated to be around 0.1 cm^2^ /V · s with I_on_/I_off_ ratio of 10^4^ to 10^5^. ZnO NWNT grown from the locally inkjet-printed Zn acetate shows similar performance of the ZnO NWNT grown from the ZnO quantum dot seeds. The ZnO NWNT did not exhibit clean saturation regime, possibly due to the increased carrier scattering by complex NW network path, large surface area, and grain boundaries at NW junctions [13]. The complex ZnO nanowire network active layer connecting the source and drain electrodes are composed of series percolation network of micron-long nanowires connected together by forming junctions during the NW growth. Since each nanowire has its own crystalline domain, the complete nanowire path that is composed of several nanowires acts as polycrystalline semiconductor [13,15]. Besides, this kind of vertically connected nanowire network may have poor associated electrostatics because some portions of the vertical nanowires lie further away from the gate and therefore experience less electric field and thus less modulation. It is believed that optimizing the nanowire slant angle by controlling the seed density and reducing the number of junctions of nanowires may improve the device performance [13]. To further improve the transfer characteristics, plasma hydrogenation or a polymer coating that can passivate surface defects and therefore restore the intrinsic properties [16] should be implemented.

**Figure 4:**
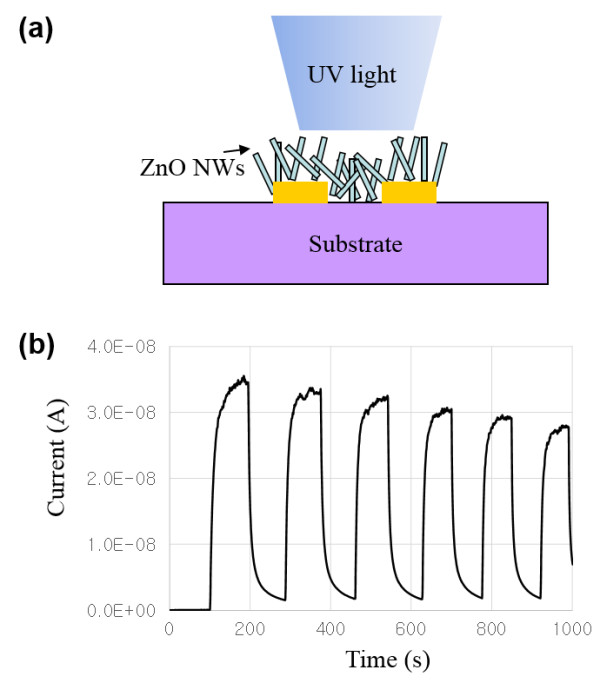
**ZnO nanowire network UV detector demonstration. (a)** Schematic illustration of the UV sensors. **(b)** Transient photoinduced current measurement under UV light with a fixed bias of 1 V. For UV illumination, a UV lamp with the center wavelength at 365 nm is turned on and off alternatively for every 100 s.

ZnO is a good candidate material for the UV detector with a bandgap of 3.2 eV. It has been proposed that the oxygen molecules adsorbed on the ZnO surface extract free electrons from doped ZnO and create a depletion layer with low conductivity which reduces the overall conductivity and, in contrast, when the ZnO is exposed to UV light, electron–hole pairs are generated and the adsorbed oxygen ions turn back into oxygen molecules as they recombine with the holes while the remaining electrons contribute to the increase in the conductivity [14,17]. Having a high surface-to-volume ratio, ZnO NW is an appropriate material for a UV sensor with high sensitivity. Figure 4a is a schematic diagram for ZnO nanowire network UV sensor locally grown on the inkjet-printed Zn acetate ink pattern. The basic structure of the ZnO UV sensor is same with the field effect transistor but without back gate. Figure 4b is the photocurrent measurement under repeated UV lamp illumination (center wavelength at 365 nm, turned on and off alternatively for every 100 s) at room temperature with 1-V external bias. The rising and decay times are estimated to be 20 to 40 s. The on/off ratio between two currents under dark and illuminating conditions are much bigger (>20) than the previously reported UV sensors. This may be because the local patterned growth of ZnO nanowires reduced the leakage current between two electrodes.

## Conclusions

We introduce a direct selective ZnO nanowire array growth on the inkjet-printed Zn acetate patterning. Zn acetate printing can completely remove the frequent clogging problems in nanoparticle or nanowire inkjet printing process. Compared with the conventional nanowire-based electronics fabrication process which is very time consuming, expensive, and environmentally unfriendly, and only a very low yield is achieved through the multiple steps, our proposed method can greatly reduce the processing lead time and simplify the nanowire-based nanofabrication process by removing multiple steps for growth, harvest, manipulation/placement, and integration of the nanowires. This process is further successfully applied to the fabrication of ZnO network transistors and UV sensor by making ZnO nanowire array network on the desired metal pattern to confirm its applicability in device fabrication.

## Abbreviations

DOD: Drop-on-demand; IC: Integrated circuit; Id: Drain current; Ig: Gate current; Is: Source current; PEI: Polyethylenimine; NWNT: Nanowire network transistors; μFE: Field effect mobility; UV: Ultraviolet; Vd: Drain voltage; Vg: Gate voltage; Vs: Source voltage; ZnO: Zinc oxide.

## Competing interests

The authors declare that they have no competing interests.

## Authors’ contributions

SH, JK, HL, and JY carried out the experiments and drafted the manuscript. SSL and SHK supervised the project and participated in the design of the study and analysis of its results. All authors read and approved the final manuscript.

## References

[B1] Ko SH Chung J Pan H Grigoropoulos CP Poulikakos D Fabrication of multilayer passive and active electric components on polymer using inkjet printing and low temperature laser processing Sensors Actuators A 2007 8 161 168 10.1016/j.sna.2006.04.036

[B2] Wang JZ Zheng ZH Li HW Huck WTS Sirringhaus H Dewetting of conducting polymer inkjet droplets on patterned surfaces Nat Mater 2004 8 171 176 10.1038/nmat1073 14991019

[B3] Sirringhaus H Shimoda T Inkjet printing of functional materials MRS bull 2003 8 802 10.1557/mrs2003.228

[B4] Chung J Ko S Bieri NR Grigoropoulos CP Poulikakos D Conductor microstructures by laser curing of printed gold nanoparticle ink Appl Phys Lett 2004 8 801 10.1063/1.1644907

[B5] Ko SH Pan H Grigoropoulos CP Luscombe CK Fréchet JMJ Poulikakos D All-inkjet-printed flexible electronics fabrication on a polymer substrate by low-temperature high-resolution selective laser sintering of metal nanoparticles Nanotechnology 2007 8 345202 10.1088/0957-4484/18/34/345202

[B6] Redinger D Molesa S Yin S Farschi R Subramanian V An ink-jet-deposited passive component process for RFID IEEE Trans Electron Dev 1978 8 51

[B7] Noh Y-Y Cheng X Sirringhaus H Sohn JI Welland ME Kang D Ink-jet printed ZnO nanowire field effect transistors Appl Phys Lett 2007 8 043109 10.1063/1.2760041

[B8] Wu J-T Hsu SL-C Tsai M-H Liu Y-F Hwang W-S Direct ink-jet printing of silver nitrate–silver nanowire hybrid inks to fabricate silver conductive lines J Mater Chem 2012 8 15599 15605 10.1039/c2jm31761c

[B9] Ko SH Lee D Hotz N Yeo J Hong S Nam KH Grigoropoulos CP Digital selective growth of ZnO nanowire arrays from inkjet-printed nanoparticle seeds on a flexible substrate Langmuir 2012 8 4787 4792 10.1021/la203781x 22126367

[B10] Greene LE Law M Goldberger J Kim F Johnson JC Zhang Y Saykally RJ Yang P Low-temperature wafer-scale production of ZnO nanowire Angew Chem Int Ed 2003 8 3031 3034 10.1002/anie.200351461 12851963

[B11] Law M Greene LE Johnson JC Saykally R Yang P Nanowire dye-sensitized solar cells Nat Mater 2005 8 455 459 10.1038/nmat1387 15895100

[B12] Ko SH Chung J Hotz N Nam KH Grigoropoulos CP Metal nanoparticle direct inkjet printing for low-temperature 3D micro metal structure fabrication J Micromech Microengr 2010 8 125010 10.1088/0960-1317/20/12/125010

[B13] Ko SH Park I Pan H Misra N Rogers MS Grigoropoulos CP Pisano AP ZnO nanowire network transistor fabrication on a polymer substrate by low-temperature, all-inorganic nanoparticle solution process Appl Phys Lett 2008 8 154102 10.1063/1.2908962

[B14] Yeo J Hong S Wanit M Kang HW Lee D Grigoropoulos CP Sung HJ Ko SH Rapid, one‒step, digital selective growth of ZnO nanowires on 3D structures using laser induced hydrothermal growth Adv Funct Mater 2013 8 3316 3323 10.1002/adfm.201203863

[B15] Gao P Brent JL Buchine BA Weinstraub B Wang ZL Lee JL Bridged ZnO nanowires across trenched electrodes Appl Phys Lett 2007 8 142108 10.1063/1.2794417

[B16] Park WI Kim JS Yi G Bae MH Lee HJ Fabrication and electrical characteristics of high-performance ZnO nanorod field-effect transistors Appl Phys Lett 2004 8 5052 10.1063/1.1821648

[B17] Hong S Yeo J Manorotkul W Kwon J An G Ko SH Low-temperature rapid fabrication of ZnO nanowire UV sensor array by laser-induced local hydrothermal growth J Nanomater 2013 8 246328

